# The intention of college students to use electronic cigarettes: A study based on the theory of innovation diffusion

**DOI:** 10.18332/tid/185644

**Published:** 2024-03-15

**Authors:** Wenjing Zou, Xiaowen Wang, Nongnong Yang, Xiaoqing Ni, Zhaoyang Zhao, Runtang Meng, Haiyan Ma

**Affiliations:** 1 School of Public Health, Hangzhou Normal University, Hangzhou, China

**Keywords:** electronic cigarette, innovation diffusion, intention to use, influencing factors

## Abstract

**INTRODUCTION:**

The purpose of this study is to examine the use of electronic cigarettes (e-cigarettes) among college students in Hangzhou, and to analyze the influencing factors of their intention to use e-cigarettes.

**METHODS:**

Using a stratified cluster sampling method, 775 students from two universities in Hangzhou were selected for an on-site questionnaire survey from March to April 2022. Adjusted logistic regression analysis was conducted on the influencing factors of use intention, based on innovation diffusion theory.

**RESULTS:**

Within our sample of college students, 16.5% of students had tried e-cigarettes; 6.32% had used e-cigarettes in the past month, and 8.0% had the intention to use e-cigarettes. There were significant differences in willingness to use e-cigarettes among different genders, economic status, smoking status of close friends around them, and their own use of tobacco and alcohol (p<0.05). The logistic regression model showed that the observability of e-cigarettes (AOR=1.28; p<0.05), personal factors (AOR=1.39; p<0.05), and social systems (AOR=1.63; p<0.05), were all influencing factors of intention to use e-cigarettes.

**CONCLUSIONS:**

College students in Hangzhou have a high intention to use e-cigarettes, and the impacts of the product itself, individual characteristics and the living environment are crucial. It is necessary to strengthen the promotion of tobacco knowledge at the social and family levels to reduce the occurrence of vaping.

## INTRODUCTION

In 2020, globally, the age-standardized prevalence of smoking among people aged ≥15 years was 22.3%, down from 32.7% in 2000^1^. Smoking can harm health, which has been confirmed through many studies. There is considerable research evidence that smoking is an important risk factor for respiratory diseases, malignant tumors, cardiovascular and cerebrovascular diseases, diabetes, and other diseases^2^. Every year, approximately 7 million people die from diseases caused by smoking^3^. Despite a downward trend, high tobacco usage rates still indicate the necessity of tobacco control.

Electronic cigarettes (e-cigarettes) are alternative, non-combustible tobacco products, mainly composed of e-liquid, power supply, heating system, and filter nozzle^4^. They generate an inhalable aerosol containing nicotine, flavors, propylene glycol, and vegetable glycerin. Through heating and atomization, and after use, people will experience similar effects as smoking cigarettes^5^. Vaping refers to the act of inhaling steam generated by electronic cigarettes or similar devices. It is often promoted as a ‘less harmful’ alternative to traditional smoking, gradually entering the lives of young students^6^. Since 2014, e-cigarettes have become the most commonly used nicotine products among young adults^7^.

The published evidence indicates that usage of nicotine e-cigarettes increases the risks of adverse health outcomes, including addiction, poisoning, toxicity from inhalation (including seizures), and lung injury^8^. Meta-analyses of 25 longitudinal studies found strong evidence that young never smokers and non-smokers who use e-cigarettes are about three times as likely as non-users to start smoking tobacco and to become regular smokers^9^. In short, e-cigarettes not only attract young people to use cigarettes but also have adverse effects on their physical and mental health and growth^10^.

An important aspect of the information surrounding e-cigarettes resides in the nature of their description as a technological innovation^11^. For example, e-cigarettes are often described in advertising as being more environmentally friendly, more satisfying, safer, or more socially acceptable than cigarettes. Because of this, we turned to the substantial body of work on the Roger’s Innovation Diffusion Theory (IDT). This theory has been used widely to examine the manner in which new products, tools, and ideas spread, and either have become widely adopted or not.

Rogers^12^ believes that diffusion is the process of spreading innovative things or ideas through certain channels among members of the social system over a period of time. At the same time, different members of the social system will not adopt an innovation at the same speed. In addition to the dissemination structure, the characteristics of innovation itself also affect the speed at which people adopt new products. Specifically, when users decide whether to adopt specific new technologies/media, they usually consider four aspects: innovation attributes (relative superiority, compatibility, ease of use, trial ability, observability), personal factors, communication channels, and social systems^13^.

For many years, innovation diffusion theory has been used to explain the spread of new ideas and practices in various environments^14^. It is often used in many other fields such as health promotion and disease prevention^15,16^, because it can help explain human behavior and provide guidance for the design of intervention measures to change behavior^17^.

As an innovation, e-cigarettes are quite popular in terms of relative advantages (possible use in places where smoking is restricted), compatibility (meet public standards or needs like other innovative products), ease of use (ease of operation), trialability (degree of feasibility), and observability (degree of impact and results that can be seen by others). Considering the specificity of electronic cigarette products as having a negative impact, the study also incorporated the factor of perceived risk.

Decades of research at the national, state, and local levels have documented how tobacco prevention mass media campaigns have contributed to the significant declines in tobacco use among young people^18^. These campaigns are thought to help change tobacco use behavior by helping to change knowledge, attitudes, beliefs, and norms around tobacco^18^.

Such knowledge will be important to develop school policies and evidence-based programs in preventing and reducing youth vaping. Based on the innovation diffusion theory, we assume that there may be a correlation between certain characteristics of electronic cigarette products (such as observability) and the willingness of college students to use electronic cigarettes.

Despite continuous progress in China’s tobacco control work, the smoking rate among Chinese people remains high. The Healthy China 2030 Plan outlines a clear goal of reducing the smoking rate of people aged ≥15 years in China to 20% by 2030^19^. Therefore, it is necessary to study the factors influencing the willingness to use e-cigarettes among college students, which can provide new ideas and measures to help achieve this goal.

This study examines the basic situation of college students’ awareness and use of e-cigarettes and explores possible factors that affect their willingness to use e-cigarettes. At the same time, from the dimensions of IDT, it provides theoretical guidance and a practical basis for subsequent intervention of e-cigarette smoking among college students. Furthermore, this study also reports on the observation of e-cigarettes among college students in Hangzhou and their exposure to the spread of e-cigarettes.

## METHODS

### Participants

This cross-sectional study used a stratified cluster sampling method to conduct a questionnaire survey on students from two universities in Zhejiang Province (one is a four-year undergraduate school and the other is a three-year vocational school). The research of Jin et al.^20^ shows that the current usage rate of electronic cigarette among college students in Zhejiang Province is 7.58%.

We used the sample size calculation formula:


$$n=\frac{Z_{0.05}^{2}\times p(1-p)}{\delta^{2}}$$

where *Z*=1.96 when the confidence level is 95%, *p* is the probability value, *δ* is the allowable error taken as 0.3*p*, giving a sample size *n* of about 520. Considering the occurrence of no response phenomenon, the sample size should not be less than 600 people.

This survey was conducted from March to April 2022, and students voluntarily participated through class notifications. The survey questionnaire is distributed and collected by the investigator on the spot to ensure its collection. This study has been approved by the Scientific Research Ethics Committee of Hangzhou Normal University (batch number: 20190100).

### Instruments

*Demographics information*

This study collected data through a field survey in 2022. The survey adopts a uniformly designed data collection table, which is a questionnaire. The questionnaire is mainly divided into three parts, including: basic information of the survey subjects, awareness and exposure to e-cigarettes, and a survey of e-cigarette usage intention. Among them, basic information included gender (male, female), grade (freshman, sophomore, junior, senior), major (Arts or Science and Engineering), monthly income (RMB, Chinese Renminbi) as disposable pocket money (<1000, 1000–2000, >2000), smoking situation of parents (yes, no, past one year), close friends who smoke (≥1, none), tobacco use (yes, no), and alcohol use (yes, no).

*Use and intention of electronic cigarettes*

The current use of e-cigarettes among college students was assessed by asking: ‘Have you ever tried e-cigarettes? (yes/no)’ and ‘How many days have you used e-cigarettes in the past 30 days? (0, 1–2, and >2 days)’. The behavioral intention of use is referred to as the learner’s choice whether to continue using e-cigarettes^21^. The intention of using e-cigarettes in the future was assessed using a Likert 5-point (1–5) rating system based on the three questions: ‘I will use e-cigarettes if my friend gives it to me’, ‘I will use e-cigarettes if an e-cigarettes enterprise or salesperson gives it to me free of charge’, and ‘I think I will use e-cigarettes in the next 12 months’. If any one question has a score ≥4 points, there is an intention to use electronic cigarettes.

*Electronic cigarette awareness*

The knowledge of the college students about e-cigarettes was measured with the questions: ‘Do you know what e-cigarettes look like?’ (yes/no) and ‘Do you know that e-cigarettes are a new type of product that is driven by batteries and forms smoke for inhalation after heating the e-cigarette liquid in the smoke cartridge through the electronic nicotine delivery system?’ (yes/no).

*Electronic cigarette transmission exposure*

Whether college students have recently been exposed to the spread of e-cigarette products was assessed by asking: ‘Have you seen people smoking e-cigarettes on TV, videos, or movies in the past 30 days?’ (yes/no) and ‘Have you seen advertisements or promotions of e-cigarettes products on the Internet or at e-cigarette outlets in the past 30 days?’ (yes/no).

*Scale of influencing factors on willingness to use electronic cigarettes*

The survey section on willingness to use is based on Rogers’ innovation diffusion theory^13,21^, which will involve 9 independent variables, namely relative superiority, compatibility, ease of use, probability, observability, personal factors, communication channels, social systems, and perceived risk (detailed information can be found in the Supplementary file Table 1). The willingness to use e-cigarettes is the dependent variable. All variables were assessed using a 5-point Likert scale: 1=strongly disagree, 2=disagree, 3=uncertain, 4=agree, 5=strongly agree. The survey subjects chose the option that best suited their feelings based on their understanding and feelings about e-cigarettes. An increasing score indicates that the respondents have a higher sense of identification with the corresponding characteristics of electronic cigarettes.

A total of 775 questionnaires were collected in this survey, with no missing items. The overall Cronbach’s alpha was 0.919, indicating that the questionnaire has good reliability and the collected data are reliable (Cronbach’ alpha coefficient ranges 0–1, with a value ≥0.7 indicating that the reliability of the scale is good).

### Analytical strategy

In this study, R version 4.2.2 was used to analyze the collected data. Frequencies and descriptive statistics of participant sociodemographic characteristics and their awareness, exposure, and use of e-cigarettes, were examined. Independent sample t-tests, analysis of variance, and chi-squared tests were used to measure differences in willingness to use e-cigarettes among respondents of different demographic characteristics. Based on the single factor analysis above, we determine whether to include it as a control factor in the subsequent model analysis. Finally, an adjusted logistic regression analysis was conducted between the independent and dependent variables based on the innovation diffusion theory, from which adjusted odds ratios (AORs) and 95% confidence intervals (95% CIs) were calculated. A p<0.05 (two-tailed) was considered statistically significant (The assignment of variables is shown in the Supplementary file Table 2).

## RESULTS

### Basic demographic characteristics

The sample size of this survey was 775, of which 33.94% (n=263) were males and 66.06% (n=512) were females; 40.80% (n=316) were freshmen, 36.52% (n=283) had monthly living expenses between 1500 and 2000 RMB, 51.23% (n=397) had at least one friend smoking by their side, 9% (n=70) smoked, and 41.50% (n=322) drank alcohol. Details of the participant characteristics are shown in Table 1.

**Table 1:** Basic demographic characteristics of university students, Hangzhou, China, 2022 (N=775)

*Characteristics*	*n*	*%*
**Overall**	775	100
**Sex**		
Male	263	33.9
Female	512	66.1
**Grade**		
Freshman	316	40.8
Sophomore	209	27.0
Junior	173	22.3
Senior	77	9.9
**Major**		
Arts	407	52.5
Science and Engineering	368	47.5
**Income** (RMB)		
<1000	87	11.2
1000–2000	510	65.8
>2000	178	23.0
**Father smoking**		
Yes	412	53.2
No	284	36.6
Past one year	79	10.2
**Mother smoking**		
Yes	12	1.6
No	758	97.8
Past one year	5	0.6
**Friends smoking**		
≥1	397	51.2
None	378	48.8
**Tobacco use**		
Yes	70	9.0
No	705	91.0
**Alcohol use**		
Yes	322	41.5
No	453	58.5

RMB: 1000 Chinese Renminbi about US$160.

### Intention to use e-cigarettes

Among the participants, 16.5% (n=128) had tried e-cigarettes; 2.5% (n=19) had used e-cigarettes in 1–2 days in the past 30 days; and 8.0% (n=62) had a willingness to use e-cigarettes.

### Intention to use e-cigarettes with different demographic characteristics

The results of the analysis of the differences in intention to use e-cigarettes with different demographic characteristics are shown in Table 2. Among them, willingness to use e-cigarettes among men was significantly higher than among women (p<0.05). Students with higher monthly income showed significantly higher willingness to use e-cigarettes than those with low income (p<0.05). Among friends with good relationships, the willingness to use e-cigarettes with at least one person smoking (including cigarettes and e-cigarettes) was significantly higher than that of non-smokers (p<0.05). The willingness to use e-cigarettes with their own smoking and drinking experiences was significantly higher than those with non-smoking and drinking habits (p<0.05). No significant differences in willingness to use e-cigarettes were found in other demographic characteristics in this study. The main results are shown in Table 2.

**Table 2:** Analysis of differences in the intention to use e-cigarettes under different demographic characteristics among university students, Hangzhou, China, 2022 (N=775)

*Variable*	*Intention to use e-cigarettes*	*t/χ* *^2^*	*p*
**Overall**	62		
**Sex**			
Male	39	15.495	0.000
Female	23		
**Grade**			
Freshman	29	7.073	0.070
Sophomore	9		
Junior	14		
Senior	10		
**Major**			
Arts	38	1.716	0.190
Science and Engineering	24		
**Income** (RMB)			
<1000	5	18.762	0.000
1000–2000	29		
>2000	28		
**Father smoking**			
Yes	40	3.813	0.149
No	16		
Past one year	6		
**Mother smoking**			
Yes	1	0.439	0.803
No	61		
Past one year	0		
**Friends smoking**			
≥1	54	33.164	0.000
None	8		
**Tobacco use**			
Yes	37	203.73	0.000
No	25		
**Alcohol use**			
Yes	49	37.329	0.000
No	13		

RMB: 1000 Chinese Renminbi about US$160.

### Awareness of and exposure to e-cigarettes among groups with different usage intentions

Among the survey respondents, 80.13% (621) had some understanding of e-cigarettes, and 72.26% (560) of college students had exposure to the spread of e-cigarette products. The chi-squared test results showed that there were significant differences in the cognitive of and exposure to e-cigarettes between the two groups of college students with and without willingness to use e-cigarettes. The main results are shown in Table 3.

**Table 3:** The association between e-cigarette awareness and exposure with the intention to use e-cigarettes among university students, Hangzhou, China, 2022 (N=775)

	*Intention to use e-cigarettes*		*χ2*	*p*
	*Willing*	*Unwilling*		
**Awareness**				
Yes	59	563	8.4521	0.004
No	3	150		
**Exposure**				
Yes	55	505	8.2289	0.004
No	7	208		

### Analysis of influencing factors on willingness to use e-cigarettes

According to the collected data, the respondents scored 6.54 ± 3.25 for their perception of the observability of electronic cigarettes (displayed through four questions, with a total score of 20), 5.73 ± 2.43 for personal factors (displayed through three questions, with a total score of 15), and 5.19 ± 2.38 for social systems (displayed through three questions, with a total score of 15). More variable scores can be found in Supplementary file Table 2.

By controlling for variables with significant differences in willingness to use e-cigarettes in demographic characteristics, a logistic regression analysis was conducted on 9 variables and willingness to use e-cigarettes to obtain Model 1. The results showed that observability (AOR=1.28; 95% CI: 1.04–1.59, p<0.05), personal factors (AOR=1.38; 95% CI: 1.08–1.78, p<0.05), and social system (AOR=1.63; 95% CI: 1.27–2.12, p<0.01) had statistically significant effects on willingness to use e-cigarettes. By increasing the control variables of e-cigarette awareness and exposure to e-cigarette transmission, Model 2 was obtained. The results still showed that only observability (AOR=1.28; 95% CI: 1.04–1.59, p<0.05), personal factors (AOR=1.39; 95% CI: 1.09–1.81, p<0.05), and social system (AOR=1.63; 95% CI: 1.27–2.12, p<0.01) had statistically significant effects on the willingness to use e-cigarettes. The main results are shown in Table 4.

**Table 4:** Adjusted logistic regression controlling for demographic characteristics, awareness of e-cigarettes, and exposure to e-cigarette transmission among university students, Hangzhou, China, 2022 (N=775)

*Variables*	*β*	*SE*	*AOR (95% CI)*	*p*
**Model 1^a^**				
Observability	0.249	0.109	1.28 (1.04 – 1.59)	0.022
Personal factors	0.319	0.127	1.38 (1.08 – 1.78)	0.012
Social systems	0.487	0.130	1.63 (1.27 – 2.12)	0.000
**Model 2^b^**				
Observability	0.207	0.102	1.28 (1.04 – 1.59)	0.042
Personal factors	0.324	0.129	1.39 (1.09 – 1.81)	0.012
Social systems	0.512	0.126	1.63 (1.27 – 2.12)	0.000

a The control variables for Model 1 include gender, monthly disposable economic status, smoking status of close friends, and personal alcohol and tobacco use.

b The control variables of Model 2 are those of Model 1 but including also awareness and transmission exposure of e-cigarettes.

AOR: adjusted odds ratio.

## DISCUSSION

Our research results show that the usage rate of e-cigarettes among college students in Hangzhou is 2.5%. A cross-sectional study from Guangzhou, China showed that the usage rate of electronic cigarettes among college students in Guangzhou is about 19.7%^22^. The usage rate of electronic cigarettes in Hangzhou is relatively low, which may be related to the small sample size in this study. A study in the United States showed that 71% of people have heard of e-cigarettes and 13% have tried e-cigarettes^23^. However, the results of this study show that over 15% of college students have tried e-cigarettes, which is worrying. In addition, understanding of and exposure to the spread of electronic cigarettes undoubtedly indicate that the attraction of electronic cigarettes by college students will be a persistent problem^20^.

Like other studies, male students have significantly higher rates and intention to use e-cigarettes than female students^24^. From the monthly disposable amount, it can be seen that the economic situation also significantly affects the willingness to use e-cigarettes. The better the economic situation, the easier it is to have the willingness to use e-cigarettes, because as an innovative product, the price is relatively high. The current regulatory system surrounding e-cigarettes has led to disposable e-cigarette manufacturers providing consumers with larger and cheaper disposable e-cigarettes, and these e-cigarettes have increasingly high nicotine concentrations^25^. This indicates the importance and urgency of implementing restrictive measures on e-cigarettes. Our findings also indicate that those who have at least one person smoking (including cigarettes and e-cigarettes) around them have a higher willingness to use e-cigarettes, which can be explained by the peer effect^26^.

The results of both logistic regression models are consistent, indicating that the observability of e-cigarettes has an association with intention to use, mainly because it is believed that the benefits of e-cigarettes are obvious. It can be considered that greater observability can provide greater functionality for the public’s practicality^27^, or a level of ‘innovative results being noticed by others’, assuming that the adopter’s friends and neighbors often ask for feedback from them^21^. For example, after using e-cigarettes, one may feel less stress and feel calm overall. Or perhaps the benefits brought by e-cigarettes are obvious. Similarly, our study has shown that personal factors are also one of the main associating factors. In daily life, those who prefer to use newly launched products often have a higher willingness to use them. In addition, the social system also significantly affects the intention to use e-cigarettes. Usually, the inherent attributes and structural characteristics of society can affect the process of product innovation and diffusion, such as the smoking situation in the surrounding environment, including close people and strangers, which can subtly affect attitudes towards vaping^28^. However, concerns have been raised about the potential health risks of vaping, particularly among youth, as well as its long-term effects on respiratory health and nicotine addiction. In addition, the culture and norms of the society, as well as relevant national policies, are also important constraints on the intention to use e-cigarettes. In the regression results of Model 2, no significant results were observed regarding the cognitive and transmission exposure of e-cigarettes, while studies have shown that online transmission has a significant impact on national ratification of the Framework Convention on Tobacco Control (FCTC)^29^. More research is needed in the future to explain this difference. Overall, in addition to the personal situation (gender, economic status) of college students, the favorable use of e-cigarettes is also an important predictor of their intention to try e-cigarettes. This indicates that attitudes towards e-cigarettes, social environmental factors, social norms, and the innovative nature of e-cigarette products, will be integrated to promote the widespread use of e-cigarettes. At the same time, effective measures targeting these aspects can also effectively reduce the willingness to use e-cigarettes.

### Limitations

This study has several limitations. Firstly, the participants were only Chinese students from two universities in Hangzhou, so the generalizability of the conclusions is limited. In the future, it is necessary to conduct a better sampling method to gain a more comprehensive understanding of the latest willingness to use e-cigarettes. Secondly, the questionnaire used in this study was self-designed, and the logical and targeted nature of the questions still needs to be strengthened to improve the quality of the questionnaire, for example, retesting reliability. Thirdly, these data are self-reported and may have reporting bias. Fourthly, the high proportion of female students in the tested schools leads to an imbalanced gender ratio in the sample size included in the study, which may result in selection bias. Fifthly, there may still be confounding factors that have not been considered during the research design phase. Finally, given that our research design is a cross-sectional study, we are unable to dynamically observe changes in e-cigarette usage intention and related factors, and therefore cannot analyze causal relationships.

## CONCLUSIONS

This study provides a broad indication that the intention rate of Hangzhou university students to use e-cigarettes is relatively high. In addition to gender, economic status, and other factors, from the perspective of the innovative diffusion characteristics of e-cigarette products themselves, the observability of e-cigarettes, as well as personal factors and social systems or living environment, may promote an increase in intention to use e-cigarettes. Currently, e-cigarettes have a broader background, which is their increasing use as a major alternative to tobacco products. This contextual backdrop underscores the escalating traction of e-cigarettes among diverse demographic cohorts, including college students. Combining the survey results, it can be seen that the dissemination exposure and public awareness of the product clearly indicate active electronic cigarette product marketing plays a significant role in increasing the willingness to use electronic cigarettes. Currently, e-cigarettes provide additional options for current tobacco users to continue to smoke. Although the moral sense of a smoking ban dominates on campus, it is still necessary to strengthen the promotion of knowledge about the hazards of e-cigarettes at the social and family levels, and reduce the occurrence of attempting to smoke e-cigarettes^30^. Electronic cigarettes still belong to innovative products. Based on the IDT, this study explored measures to curb the use of electronic cigarettes from the perspective of the influencing factors of the product itself, such as reducing the positive promotion of electronic cigarette products. At the same time, it provides new ideas for research methods.

## Supplementary Material



## Data Availability

The data supporting this research are available from the authors on reasonable request.
