# Supplementary material for: The intention of college students to use electronic cigarettes: A study based on the theory of innovation diffusion
Source: Tob Induc Dis. 2024 Mar 15;22:10.18332/tid/185644. doi: 10.18332/tid/185644 (PMC10941754; doi:10.18332/tid/185644)
Supplement: Supplementary file 1 [file TID-22-53-s1.pdf]

Table 1. A survey on willingness to use electronic cigarettes based on Rogers innovation diffusion theory among college students in Hangzhou, China 2022(N=775)

Topic	Strongly disagree	Disagree	Indeterminacy	Agree	Strongly agree
**D1.** Using e-cigarettes can help me better integrate into the social environment around me.					
**D2.** Electronic cigarettes can help me quit smoking better.					
**D3.** The harm of electronic cigarettes is smaller than that of cigarettes.					
**D4.** The addiction of electronic cigarettes is lower than that of cigarettes.					
**D5.** I think using e-cigarettes aligns well with my values.					
**D6.** The popularization of electronic cigarettes is in line with the development of the times.					
**D7.** E-cigarettes come in various flavors that can meet my needs					
**D8.** Learning to use e-cigarettes is very easy for me					
**D9.** I can easily obtain the electronic cigarette information I want to know					
**D10.** I can easily purchase/obtain e-cigarettes					
**D11.** Electronic cigarettes can be charged using USB					
**D12.** All e-cigarette sales points I come into contact with can provide trial smoking services					
**D13.** I have tried e-cigarettes with friends before					
**D14.** I hope to have more opportunities to try e-cigarettes for free or at a low price					
**D15.** After using e-cigarettes, I can understand the benefits of e-cigarettes very well					
**D16.** I can easily explain the					

benefits of e-cigarettes to others					
**D17.** I think the benefits of e-cigarettes are obvious					
**D18.** After using e-cigarettes, I feel less pressure and feel very calm					
**D19.** Using e-cigarettes can be harmful to my health					
**D20.** Electronic cigarettes can make me addicted					
**D21.** Using e-cigarettes can be harmful to people around me					
**D22.** If I use e-cigarettes, my parents will criticize me					
**D23.** If I use e-cigarettes, some of my friends may distance me					
**D24.** Using e-cigarettes will increase my monthly expenses					
**D25.** In daily life, I enjoy using newly launched products					
**D26.** I have a favorable impression of smoking as a behavior					
**D27.** I have tried e-cigarettes before and the experience was good					
**D28.** I can see e-cigarette related advertisements or promotions on mass media (including posters, flyers, television, and the internet) at least once a week					
**D29.** People around me who have used e-cigarettes recommend me to use e-cigarettes					
**D30.** I hope e-cigarette salespeople can introduce me to the relevant information about e-cigarettes					
**D31.** People around me all use e-cigarettes					
**D32.** Electronic cigarettes are suitable for the culture and norms of my social					

environment					
**D33.** I believe that national policies support the development of electronic cigarettes					
**D34.** If my friend gives me an e-cigarette, I will use it					
**D35.** If any e-cigarette company or salesperson gives me e-cigarettes for free, I will use them					
**D36.** In the next 12 months, I believe I will use e-cigarettes					

Table 2. The assignment of variables in the study of intention to use e-cigarettes based on Rogers innovation diffusion theory among university students, Hangzhou, China 2022 (N=775)

Variables	Details
Intention to use e-cigarettes	
	1=Yes,Willing to use electronic cigarettes
	0=No,Not willing to use e-cigarettes
Age	
	1=male
	2=female
Monthly disposable change	
	1=Up to 1000￥
	2=1000 to 2000￥
	3=Greater than 2000￥
Father smoking or not	
	1=Smoke
	2=No Smoking

	3=Previously smoked, but has stopped smoking for more than a year
Mather smoking or not	
	1=Smoke
	2=No Smoking
	3=Previously smoked, but has stopped smoking for more than a year
Smoking situation of friends	
	1=At least one person smokes
	2=No one smokes
Smoking cigarettes	
	1=Yes,Used cigarettes within the past 30 days
	2=No, Haven't used cigarettes in the past 30 days
Alcohol intake	
	1=Yes, Have consumed alcohol within the past 30 days
	2=No,Haven't consumed alcohol within the past 30 days

© **2024 Zou W. et al.**
